# Enhanced Electrothermal Properties of Core–Sheath Lignin-Derived Carbon Nanotube Yarns with UHMWPE Insulation

**DOI:** 10.3390/polym17040537

**Published:** 2025-02-19

**Authors:** Hongmei Dai, Chao Jia, Zexu Hu, Senlong Yu, Hengxue Xiang, Xuefen Wang, Meifang Zhu

**Affiliations:** State Key Laboratory of Advanced Fiber Materials, College of Materials Science and Engineering, Donghua University, Shanghai 201620, China; 1209738@mail.dhu.edu.cn (H.D.);

**Keywords:** electrothermal fabric, carbon nanotubes, core–sheath yarns, UHMWPE

## Abstract

A critical challenge in wearable electrothermal textiles is achieving effective insulation while maintaining sheath flexibility, which is essential for enhancing the mechanical properties and durability of conductive materials under everyday conditions, such as washing, stretching, and twisting. In this work, we employ a coaxial tubular braiding technique to coat a high-conductivity carbon nanotube (CNT) yarn with a high-strength insulation layer made of ultra-high-molecular-weight polyethylene (UHMWPE) multifilaments, resulting in a core–sheath-structure CNT yarn with excellent electrothermal performance. By adjusting the number of UHMWPE multifilaments and the sheath braiding angle, we achieve high flexibility, high tensile strength, and abrasion and wash resistance, as well as improved electrical stability for the CNT yarns. Additionally, the CNT yarns with an insulation layer effectively prevent short-circuiting during use and achieve superior thermal management, with a significant increase in steady-state temperature under operational conditions, exhibiting significant potential for applications in wearable electronic devices.

## 1. Introduction

In the field of wearable electrothermal clothing and textile electronics, conductive fibers and yarns play a pivotal role, functioning as conductors, Joule heating elements, and integral textile components [1,2,3]. Various conductive materials, including metal nanoparticles and nanowires [4], carbon nanotubes (CNTs) [5,6], graphene [7], two-dimensional transition metal carbides and nitrides (MXenes) [8,9], conductive polymers [10], and their composites [11], have been extensively studied and applied. These materials enable the seamless integration of electrothermal elements with conventional fibers and yarns. However, despite these advancements, several technical challenges remain. A key issue is achieving a balance between reliability and flexibility when connecting materials with mismatched mechanical properties. Additionally, electronic components must be equipped with flexible non-conductive encapsulations that ensure adequate electrical insulation while withstanding everyday wear and tear, such as washing, stretching, and twisting [12,13,14,15]. Traditional insulation methods, although effective at protecting conductive yarns, often reduce the flexibility of these materials and exacerbate the issue of modulus mismatch in textile applications, limiting their practical use.

To address these challenges, researchers have proposed various methods to improve the insulation and mechanical properties of conductive yarns. One such method is direct polymer dip-coating, which provides some insulation but often compromises both conductivity and flexibility [16,17,18]. For instance, in Li’s study, increasing the PVA concentration from 1 to 7 wt% led to a continuous decrease in the electrical conductivity of CNT/PVA-coated yarns, with an estimated reduction of approximately 70%, from about 950 S/cm to 300 S/cm [17]. Electrospun nanofiber coatings, while maintaining flexibility, lack sufficient strength to significantly improve the material’s overall performance [19,20]. Yin’s research demonstrated that electrospinning silk nanofibers onto CNT yarn preserved conductivity and flexibility, but reduced tensile strength, with specific stress decreasing from 123 cN/tex to 16 cN/tex [20]. Continuous hollow spindle spinning, which encases conductive yarns in a continuous insulation layer, offers improved mechanical protection and performance retention [21]. However, in practical use, the insulating layer may expose the conductive core during bending or under stress, creating a risk of short-circuiting. Tubular braiding, another technique used in triboelectric nanogenerators (TENGs), has shown promise due to its high compressive elasticity and pressure sensitivity, achieving impressive results [22,23]. However, this method often involves larger diameters and has not yet fully resolved the issue of resistivity changes during use, which is crucial for electrothermal applications. Furthermore, these braided systems frequently incorporate silver-plated yarns as conductors, which raises concerns about coating degradation, such as cracking or peeling, under mechanical stress or repeated washing [24,25].

**Figure 1:**
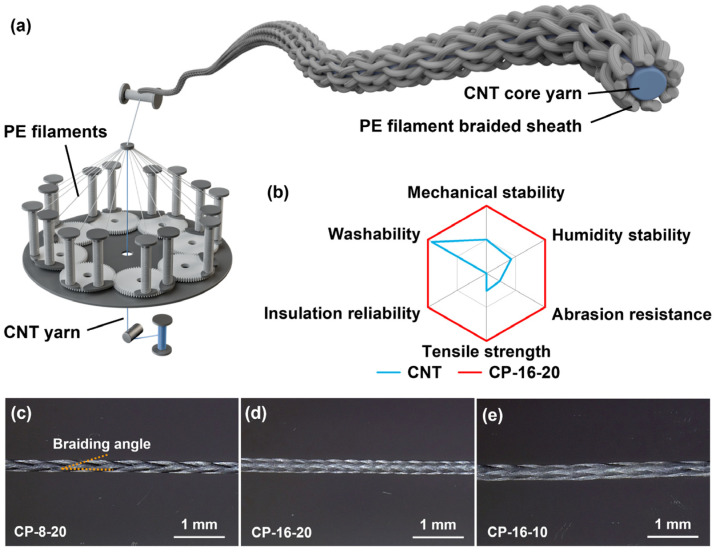
Preparation and morphology of CP yarns. (**a**) Schematic showing the preparation process of CP yarns. (**b**) Property comparison of CP yarns and CNT yarns in term of mechanical stability, humidity stability, abrasion resistance, tensile strength, insulation reliability, and washability. (**c**–**e**) EDOF images of CP-8-20, CP-16-20, and CP-16-10.

In this study, we developed a core–sheath CNT/UHMWPE yarn (CP yarn) by encapsulating CNT yarn with ultra-high-molecular-weight polyethylene (UHMWPE) filaments using a tubular braiding process (Figure 1a). The UHMWPE sheath significantly enhances durability and mechanical stability while maintaining the yarn’s lightweight, flexible, and conductive properties, making it highly effective for electrothermal applications (Figure 1b). By optimizing the braiding angle and filament count, we achieved near-complete core coverage, improving both electrical safety and Joule heat transfer efficiency. Moreover, the core–sheath yarn shows potential for innovative applications, such as embroidered components capable of generating Joule heat through near-field energy reception. This suggests promising future integration of comfort and energy efficiency in advanced textile technologies.

## 2. Experimental Section

### 2.1. Preparation of CP Yarn and Fabric

In this study, CNT yarns (25 tex) were fabricated following our previously reported floating catalyst chemical vapor deposition (FCCVD) method [26], while UHMWPE multifilaments (Spectra UF BIO25; Honeywell International Inc., Herndon, VA, USA) were commercially sourced. These two components were combined to form CP yarn, where “CP” denotes the combination of CNT yarns and UHMWPE filaments. Three variations of core–sheath yarns were fabricated using a tubular braiding technique, with CNT yarn as the core and different sheath densities achieved by adjusting the braiding parameters of the UHMWPE sheath. Sheath densities were modulated by altering the braiding angle, pitch, and the number of UHMWPE filaments, using either 8 or 16 filaments. These parameters were precisely controlled via adjustments to the active wheel and gear of a 16-slot horn gear machine, which featured a central hole for the core yarn (Figure 1a). The resulting yarns were designated as CP-8-20, CP-16-20, and CP-16-10, indicating the filament bundle count and braiding angle. The braiding angles were accurately measured using MB-Ruler on digital images obtained through extended depth of field microscopy (EDOF), determining the angle between the CP yarn axis and the UHMWPE filament axis.

### 2.2. Characterizations and Mechanical Testing

The morphology, microstructure, and folding characteristics of the CNT and CP yarns were examined using a scanning electron microscope (SEM; SU8010, Hitachi High-Technologies Corporation, Tokyo, Japan) and an extended depth of field microscope (EDOF; Leica DVM6, Leica Microsystems, Wetzlar, Germany).

The insulation performance was evaluated by torsionally twisting two identical yarns (labeled A and B) with a specified braiding density. The yarns were twisted 10 times and secured within a 5 cm testing frame. Two SourceMeters (Keithley 2450, Tektronix, Inc., Cleveland, OH, USA) were used: one applied a current to yarn A (−0.5 A to 0.5 A at a rate of 0.2 A s^−1^), while the other monitored the current and voltage in yarn B, which received no current input. The voltage response over time was recorded to assess the insulation effectiveness.

Tensile properties were tested using a tensile tester (INSTRON 5966, Instron Corporation, Norwood, MA, USA) fitted with specialized yarn grips. Specimens, each 50 cm long with a 30 cm gauge length, were tested at a rate of 10 mm min^−1^. Resistance during the tensile testing process was monitored with a digital multimeter (Keithley DMM 6500, Tektronix, Inc., Cleveland, OH, USA), and the toughness, stress–strain curves, and changes in resistance were analyzed.

Fatigue tests, including 0-degree folding, constant elongation, and 360-degree torsional fatigue, were performed using a dynamic mechanical fatigue testing machine (Electro Force 3230, Instron Corporation, Norwood, MA, USA). In the 0-degree folding test, CNT and CP yarns were vertically secured with a 6 mm gauge, and a 200D spandex filament was folded over the midpoint. The system cyclically compressed the upper fixture by 5 mm at a frequency of 5 Hz for 2500 cycles, achieving near-0-degree folding with a minimum gauge of 1 mm. For the constant elongation test, yarns were fixed at a 10 cm gauge and stretched by 1 mm at 5 Hz for 5000 cycles. The 1 mm stretch was selected, as it applies a force exceeding 2 N, which is significant for a single yarn. In the 360-degree torsional fatigue test, the lower fixture remained stationary while the upper fixture rotated counterclockwise by 180 degrees, returned to the starting position, and then rotated clockwise by 180 degrees, repeating this cycle for 100 rotations. A pre-tension of 1 N was applied before the torsional load. Throughout all tests, electrical resistance was monitored to evaluate any variations in resistance.

### 2.3. Determination of Electrothermal Properties

A 100-cycle cyclic electrothermal experiment was conducted on both CNT and CP yarns at current levels of 0 A, 0.05 A, and 0.10 A, using a Keithley source meter as a programmable power supply. During the experiments, temperature changes were monitored with a Fotric infrared thermal imager. Temperature–time (T-t) curves were generated to evaluate response time and energy efficiency during the electric heating process. These curves were categorized into three stages: heating, steady-state temperature, and cooling. Empirical formulations were used to describe the temperature increase and decay, with time constants for the heating (*τ_g_*) and cooling (*τ_d_*) phases determined through data fitting (Formulas (1) and (2)). The heat transferred by radiation and convection (*h_r+c_*) during the steady-state stage was expressed using Formula (3) [27].

(1) $$\frac{T_{t}-T_{0}}{T_{m}-T_{0}}=1-exp\left(-\frac{t}{\tau_{g}}\right)$$

(2) $$\frac{T_{t}-T_{0}}{T_{m}-T_{0}}=exp\left(-\frac{t}{\tau_{d}}\right)$$

In the aforementioned formulas, *T*_0_ represents the initial ambient temperature, while *T_m_* denotes the maximum temperature reached under steady-state conditions. *T_t_* corresponds to the temperature at a specific time (*t*), with *τ_g_* being the characteristic time constant for temperature increase (growth) and *τ_d_* the characteristic time constant for temperature decrease (decay).

(3) $$h_{r+c}=\frac{I_{C}V_{0}}{T_{m}-T_{0}}$$

In this formula, *V*_0_ represents the externally applied voltage, while *I_c_* denotes the steady-state current.

### 2.4. Durability Testing

The yarns were subjected to abrasion tests by rubbing against medium-grit abrasive paper, which wrapped around a cylindrical object with an approximate diameter of 20 mm. One end of the yarn was fixed, while a 5 g weight was attached to the other end to apply a consistent tension. The cylinder rotated at 2 rpm, with friction applied along the yarn’s axis, moving toward the weighted end.

For humidity sensitivity testing, the yarn samples were conditioned in a controlled laboratory environment, where their electrical resistance was monitored. The samples were exposed to 70% relative humidity (RH) using a Ø60 mm × 20 mm crystal humidifier in a Ø85 mm × 45 mm container, along with a humidity card. For comparison, the samples were also subjected to 10% RH using anhydrous calcium chloride in a similar container with a humidity card.

CNT yarn and three types of CP yarns were sewn onto a multifiber standard fabric using a 2 mm needle gauge. These samples were subjected to accelerated washing using a standard soap solution, following the AATCC TM 61-1A guidelines [28]. Electrical resistance and morphological changes were assessed before and after washing to evaluate durability and performance.

## 3. Results and Discussion

### 3.1. Material Structures and Morphology

The tubular braided yarn structure features fine porosity, enabling effective heat dissipation, which makes the CP yarns ideal for thermal management applications (Figure 1a). The structure also offers exceptional mechanical stability due to its interlaced configuration, effectively resisting tensile, torsional, and bending stresses, ensuring stability in various operational environments (Figure 1b). Despite its robustness, the tubular braid remains highly flexible, making it well-suited for applications requiring complex geometries or wearable technologies with a soft touch.

UHMWPE was selected as the sheath material due to its superior shear resistance, high strength-to-weight ratio, and low density, which significantly improve the yarns’ resistance to stretching, folding, twisting, abrasion, and tensile forces. The measured crystallinity of the UHMWPE multifilaments (91.4%) correlates with its mechanical robustness, while the dual melting peaks at 148.6 °C and 153.5 °C further indicate its suitability for electrothermal applications (Figure S1). The dual peaks are likely due to variations in crystalline domains formed during the manufacturing process. The braided structure of the sheath introduces pores that promote heat convection, while the material’s thickness and density continue to influence overall thermal performance. Additionally, UHMWPE’s high transparency to infrared radiation minimizes its effect on radiative heat transfer, further optimizing thermal management [29]. By arranging UHMWPE filaments into tightly packed layers, we created two- and four-layer configurations to observe the anticipated reduction in infrared transmittance as the number of filament bundles increased (Figures S2 and S3).

In typical core–sheath yarns, braiding angles between 20° and 75° are used to balance flexibility and core coverage [30]. A braiding angle of 20° with 16 filaments was found to be optimal, providing near-complete core coverage and ensuring electrical safety while enabling efficient Joule heat transfer. To further improve flexibility, we experimented with smaller braiding angles and reduced sheath filament counts to lower coverage density. Through systematic variation in filament count and braiding angle, three configurations were selected for detailed analysis.

EDOF analysis showed that the CNT yarns have a diameter of approximately 0.18 mm (Figure S4), with all CP yarns maintaining full core coverage when straightened (Figure 1c–e). The CP-8-20 and CP-16-20 yarns, both braided at a 20° angle, have diameters of 0.34 mm and 0.39 mm, respectively. Despite doubling the filament count, the diameter of CP-16-20 increased by only 14.7% (Figure S5a,b), resulting in a 31.6% increase in cross-sectional area. This relatively modest increase in cross-sectional area suggests that larger tensile strengths can be achieved, as the additional filaments enhance the structural integrity without doubling the overall diameter of the yarn. Their pitch distances were measured at 0.98 mm and 0.48 mm, respectively. The pitch ratio of them is approximately 2:1, indicating that the increase in filament number does not significantly alter the inter-fiber spacing.

The CP-16-10 yarn, braided at a 10° angle, has a diameter of 0.45 mm, reflecting a 33.1% increase in cross-sectional area compared to CP-16-20 (Figure S5c), with a pitch distance of 0.96 mm. For the same filament count, this increase in cross-sectional area leads to a reduction in tensile strength, as the relative proportion of core material capable of resisting applied force decreases. Furthermore, larger braiding angles and higher filament counts resulted in a denser structure, with CP-16-10 exhibiting the highest flexibility, followed by CP-8-20, while CP-16-20 was the stiffest, indicating a reduction in flexibility. The increased flexibility of the CP-16-10 yarn may slightly compromise the protective role of the sheath layer, but, simultaneously, it allows for greater dynamic motion. This can prevent excessive compression of the conductive core, potentially reducing the risk of mechanical failure. Additional challenge tests are required to further validate these observations, particularly under dynamic and repeated loading conditions, to ensure the long-term stability and performance of the yarns in wearable electronic applications.

### 3.2. Mechanical, Electrical, and Electrothermal Behavior of CP Yarns

**Figure 2:**
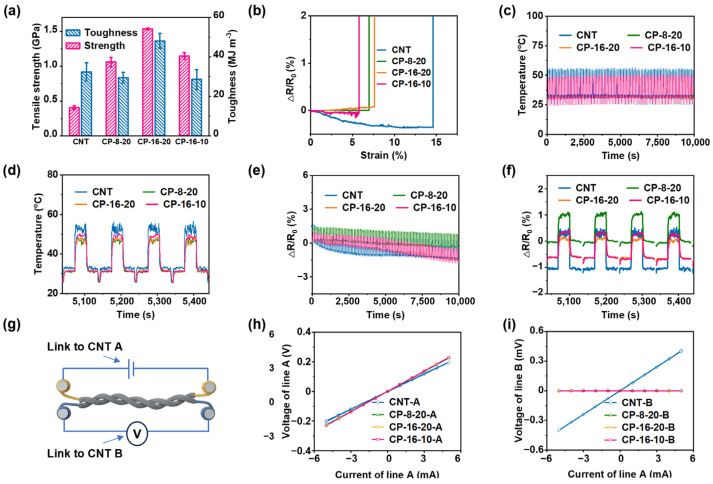
Mechanical, electrical, and electrothermal properties of CNT and CP yarns. (**a**) Tensile strength and toughness. (**b**) Resistance–strain curves during the tensile test. (**c**) Temperature–time curves in a 100-cycle electrothermal experiment at 0 A → 0.05 A → 0.10 A → 0.05 A → 0 A and (**d**) the magnified curves. (**e**) Resistance–time curves in the 100-cycle electrothermal experiment at 0 A–0.05 A–0.10 A and (**f**) the magnified curves. (**g**) Schematic of insulation performance test. (**h**) Voltage–current data graph for source meters applied to line A during the insulation performance test. (**i**) Voltage–current data graph for source meters applied to line B during the insulation performance test.

The core–sheath yarns exhibit significantly higher tensile strength compared to the CNT yarn, primarily due to the hoop stress applied by the braided sheath (Figure S6) [31,32,33]. The tensile strength increases with the sheath filament count, as seen in CP-16-20 and CP-16-10, despite CP-16-10’s smaller braiding angle. Larger braiding angles, such as in CP-16-20 and CP-8-20, create more internal space within the braided structure, allowing for greater elongation during stretching. As a result, CP-16-20, with its denser structure, demonstrates higher elongation compared to CP-16-10. In addition, CP-16-20 shows significantly enhanced toughness compared to the CNT yarns, indicating better resistance to impact and abrasion (Figure 2a).

Although CP-8-20 and CP-16-10 have higher tensile strength than the CNT yarns, they do not achieve the same level of toughness as CP-16-20, indicating that although they withstand stretching forces well, CP-16-20 provides a superior balance of strength and durability. This is consistent with the observation in Section 3.1 that a relatively smaller cross-sectional area leads to a proportionally higher tensile strength. EDOF analysis of fracture sites revealed that yarns with larger braiding angles, such as CP-8-20 and CP-16-20, exhibit tightly bonded interfaces between the CNT core and UHMWPE sheath (Figure S7). In contrast, CP-16-10, with its smaller braiding angle, shows clean separation between the core and sheath upon fracture, with the sheath appearing frayed due to recoil forces.

In terms of electrical resistance, the CNT yarns show a consistent decrease in resistance until fracture, aligning with previous studies that highlight conductivity enhancement under tension (Figure 2b) [34,35,36]. However, in CP yarns, the interaction between the insulating material in the braided sheath and the CNTs during radial compression affects conductivity [37,38]. This interaction competes with the original conductive pathways, leading to fluctuations in resistance during stretching. Among the CP yarns, CP-8-20 maintains stable resistance throughout the process, while CP-16-20 shows a slight increase, and CP-16-10 exhibits a minor decrease before fracture (Figure 2b). These findings highlight the influence of braiding density on mechanical integrity and resistance behavior. The superior toughness of CP-16-20, combined with its ability to maintain conductivity, underscores its effectiveness and suitability for applications that demand both strength and reliable electrical performance.

The electrothermal behavior of the four yarn types shows excellent repeatability and stability (Figure 2c,d). CNT yarns exhibit rapid millisecond-range rise and fall times, while CP yarns require approximately 1–2 s to reach similar thermal responses. In steady-state conditions, CP yarns show slightly lower temperatures than CNT yarns; for instance, while the CNT yarn stabilizes at 32.6 °C, CP-8-20, CP-16-20, and CP-16-10 stabilize at 31.0 °C, 31.3 °C, and 31.5 °C, respectively. At higher temperatures, the differences become more pronounced, with CNT yarn reaching about 52.5 °C, while CP yarns stabilize at lower temperatures of 46.8 °C, 46.6 °C, and 48.8 °C (Figure S8, Table S1). These temperature variations can be attributed to both macroscopic and microscopic factors. On a macroscopic level, the core–sheath structure affects heat convection, with the tighter braiding in CP yarns influencing heat transfer efficiency. The larger braiding angles in CP-8-20 and CP-16-20 contribute to greater temperature decrease due to their impact on heat convection.

On a microscopic level, several mechanisms are at play: CNTs have high thermal conductivity, enhancing heat conduction along the core, while the UHMWPE sheath has lower thermal conductivity. CNTs exhibit anisotropic heat transfer, with higher axial conductivity compared to radial conductivity due to their alignment. Interface thermal resistance between the CNT core and the UHMWPE sheath also affects the overall thermal conductivity. These factors lead to differences in heat transfer efficiency between the axial and radial directions, resulting in CP yarns showing lower steady-state temperatures compared to the CNT yarns. The combined macroscopic and microscopic influences cause the CP yarns to exhibit slightly reduced heat transfer efficiency, as evidenced by their lower steady-state temperatures.

The resistance–time analysis reveals that each current load causes a slight resistance increase accompanied by heating, which returns to its original or a slightly lower value upon cooling (Figure 2e). CNT yarns show the highest resistance increase per cycle (Figure 2f), likely due to thermal motion that expands inter-tube spacing, increasing electron transport [39,40]. This increase suggests minor structural relaxation or defect reduction, but it is more controlled in CP yarns, thanks to the radial compression exerted by the UHMWPE sheath. The sheath restricts the expansion of inter-tube spacing in the CNT core during Joule heating, leading to a more stable resistance trend.

In the insulation performance test, the voltage–current relationship for all four yarn types shows linear behavior (Figure 2g–i). For the CNT yarns, the voltage on line B increased proportionally with the input current on line A, indicating unintended current transmission due to direct contact, which reveals the lack of effective insulation. In contrast, for the three CP yarns, the voltage on line B remained at zero despite the input current on line A, demonstrating superior insulating properties.

### 3.3. Flexibility and Durability of CP Yarns

**Figure 3:**
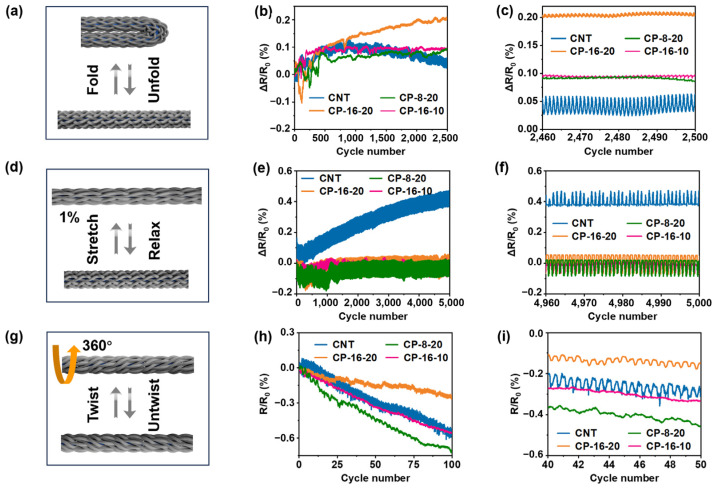
Flexibility and durability of CP yarns. (**a**) Schematic diagram of the 0-degree folding test, (**b**) resistance change rates during the folding test, and (**c**) the magnified curves. (**d**) Schematic diagram of the constant elongation fatigue test, (**e**) resistance change rates during the test, and (**f**) the magnified curves. (**g**) Schematic diagram of the 360-degree torsional fatigue test, (**h**) resistance change rates during the torsion, and (**i**) the magnified curves.

During the initial 0-degree folding tests, all CNT yarns exhibited random resistance fluctuations of approximately 0.10%. After 400 cycles, the resistance stabilized, while CP-16-20 showed a gradual increase, reaching around 0.20% by 2500 cycles (Figure 3a,b). This slight increase is likely attributed to the higher braiding density, which compresses the conductive core during folding (Figure S9). Although reducing the number of braiding filaments or adjusting the braiding angle could improve flexibility and smoothness, the denser braiding of CP-16-20 offers significant advantages. Under folding stress, CP-16-20 maintains more complete sheath coverage, minimizing the risk of exposure that may occur in more loosely braided configurations. Therefore, CP-16-20 remains a strong candidate for conductive applications, providing structural stability and enhanced protection against short circuits.

Throughout the folding cycles, resistance exhibited periodic variations, with CP yarns showing minimal fluctuations compared to the more pronounced changes in CNT yarn. In the final 50 cycles, the CNT yarns displayed regular resistance variations (~0.03%), likely due to partial short circuits during folding, whereas CP yarns maintained very minimal fluctuations (<0.01%), thanks to their insulating layers, which effectively prevented short circuits (Figure 3c).

In constant elongation fatigue tests, the CNT yarns exhibited an irreversible resistance increase, reaching 0.37% after 5000 cycles (Figure 3d,f). Each stretching cycle caused a temporary rise in resistance, which partially recovered after unloading, indicating cyclic alterations in the conductive paths. However, the overall increasing resistance trend suggests permanent sliding between individual CNTs during elongation [41,42,43]. In contrast, the CP yarns maintained stable resistance throughout the test. The UHMWPE-reinforced sheath provided structural elasticity, preventing damage and ensuring consistent performance. During stretching, the radial compression of the CNT core improved inter-tube contact, reducing resistance, which fully returned to its original value upon unloading.

In the torsional fatigue test, both CNT and CP yarns generally exhibited a decrease in electrical resistance with repeated twisting (Figure 3g–i). CNT yarns exhibited periodic resistance changes (~0.05%) due to realignment of the conductive pathway, with resistance decreasing as twisting brought CNTs closer together. In contrast, CP yarns, including CP-8-20 and CP-16-10, showed a steady decline in resistance without significant fluctuations, owing to effective radial forces that minimized CNT separation. Although CP-16-20 demonstrated robust performance and stability, its overall resistance decrease was smaller. This suggests that enhancing the flexibility of CP-16-20 could further improve its electrical performance while retaining its existing advantages.

### 3.4. Abrasion and Wash Resistance of CP Yarns

**Figure 4:**
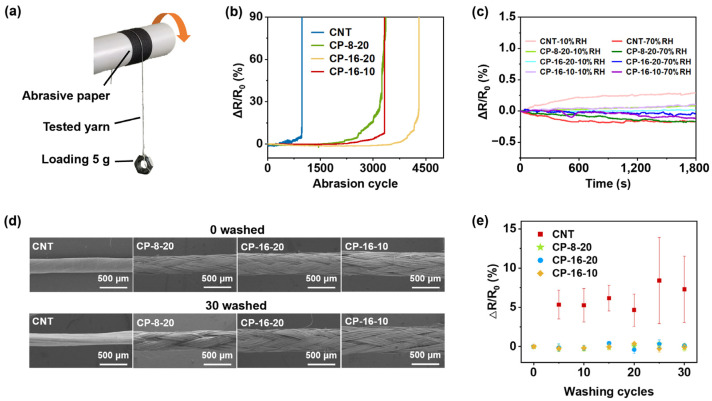
Abrasion and wash resistance of CP yarns. (**a**) Schematic of the abrasion resistance test set-up. (**b**) Resistance change rate-versus-abrasion cycle curves of the abrasion resistance test. (**c**) Resistance change rate-versus-time curves of the humidity sensitivity test. (**d**) SEM images of the CNT and CP yarns before and after washing. (**e**) Resistance change rates versus washing times.

The abrasion test revealed that CNT yarns experienced the earliest breakage, whereas CP-16-20 demonstrated superior durability, with the longest time to break (Figure 4a,b). CP-16-10 and CP-8-20 had similar breakage times. This indicates that the tighter braiding of UHMWPE and CNT yarn enhances mechanical strength and abrasion resistance, with CP-16-20 showing the best durability. The resistance change rate before breakage in CP-16-10 and CNT yarns followed a similar trend, suggesting that the sheath layer’s protective function in CP-16-10 may be compromised as the CNTs become exposed during abrasion.

In humidity sensitivity tests, CNT yarns exhibited the greatest resistance variation, with changes of approximately 0.22% at 10% RH and −0.16% at 70% RH after 10 min, increasing to 0.29% and −0.17%, respectively, after 30 min (Figure 4c). In contrast, CP yarns displayed better stability, with CP-16-20 showing the least resistance change, followed by CP-16-10 and CP-8-20. Specifically, at 10% RH, the resistance changes for CP-16-20, CP-16-10, and CP-8-20 were approximately 0.01%, 0.11%, and 0.09%, respectively, and at 70% RH, the changes were −0.03%, −0.11%, and −0.17%. The superior humidity resistance of CP-16-20 is attributed to its denser filament bundles and tighter braiding. Notably, CP-8-20, with the thinnest sheath, showed resistance changes similar to CNT yarns after 20 min at 70% RH.

Visual inspection and SEM observations showed minimal changes of the CNT yarns and CP yarns after 30 cycles of simulated washing (Figure 4d and Figure S10). CNT yarns displayed slight deformation and CP-8-20 experienced minor sheath loosening, while CP-16-20 and CP-16-10 remained largely unaffected. All CP yarns exhibited minimal changes in electrical resistance compared to CNT yarns, highlighting the effective protective performance of the core–sheath structure (Figure 4e).

### 3.5. Application Performance of CP Yarns

**Figure 5:**
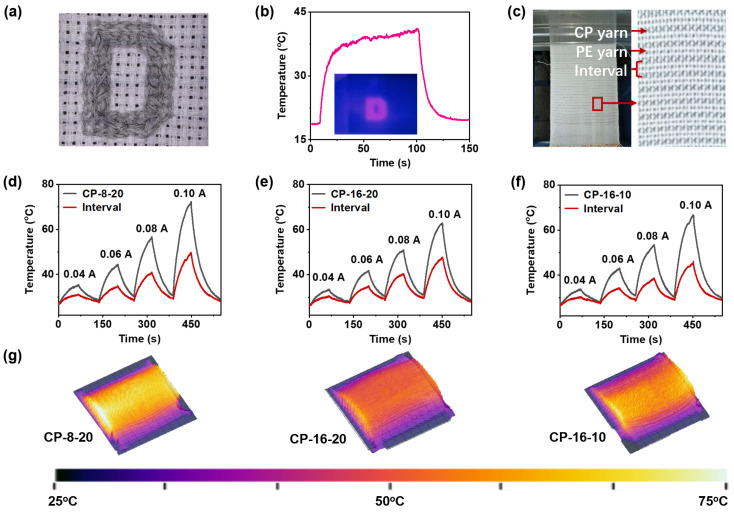
Application performance of CP yarns. (**a**) Digital image of a cross-stitch pattern featuring the letter “D”. (**b**) T-t curve and thermal image showing Joule heat conversion from near-field energy reception of the “D” cross-stitch. (**c**) Digital image of a woven fabric with CP yarns arranged in parallel at 3 mm intervals. Heating and cooling T-t curves for the woven fabrics at 0.04 A, 0.06 A, 0.08 A, and 0.10 A for (**d**) CP-8-20, (**e**) CP-16-20, and (**f**) CP-16-10, with (**g**) corresponding thermal images at 0.10 A after 1 min.

Among the three CP yarn variants, CP-16-20 was deliberately selected for stress testing under repeated bending due to its higher filament count and tightly braided structure, which maximize structural rigidity at the cost of flexibility. This intentional trade-off positions CP-16-20 as a benchmark for evaluating fatigue resistance under cyclic mechanical deformation, such as that experienced during embroidery, where dense stitching patterns impose frequent bending forces. For embroidery, 0.5 m of CP-16-20 yarn was applied to a 14-count cross-stitch fabric. A “D” letter, approximately 1.5 cm by 2.0 cm, was embroidered using full cross-stitch and backstitch techniques, forming 10 continuous loops with the core yarn ends joined (Figure 5a). Another “D” was embroidered on 40 S × 40 S/27 × 26 cotton gauze fabric using an outline stitch with five continuous loops, similarly ensuring that the core yarn ends were connected. To evaluate the near-field energy reception capability, the “D” devices were used as the receiving apparatuses, converting the induced alternating magnetic field into electrical energy for Joule heating. The devices were placed near a wireless power transmitter (WP3650T, Wupai Technology, Shenzhen, China), separated by a transparent PMMA sheet. Temperature changes were monitored using a Fotric infrared thermal imager. For woven fabric samples, three were produced using a semi-automatic loom. The warp was composed of 400D/3 UHMWPE braided yarns, with 110 threads threaded through 10 dents per centimeter (DPC) reed. The weft incorporated alternating patterns of one CP yarn and two UHMWPE braided yarns, creating a woven fabric with CP yarns spaced 3 mm apart.

In dense embroidery, CP-16-20 maintains its appearance, with patterns remaining flat and securely adhered to the fabric surface. Combining mechanical, electrical, and practical advantages, it is well-suited for compact wearable devices. The embroidered circuit structure effectively absorbs and senses Joule heat, as demonstrated by the temperature–time (T-t) curve and thermal imaging in Figure 5b, the temperature rises from approximately 19 °C to 36 °C within just 15 s. CP-16-20’s performance in these demanding tests suggests that more flexible yarns may perform even better in practical applications.

The sparse “D” embroidery design highlights the ability of this small-scale embroidery to rapidly generate Joule heat when positioned near a coil (Video 1). This design, which wirelessly receives energy and produces heat without physical connections to electrodes or power sources, exemplifies innovation in creating compact and lightweight electronic textiles. The woven fabric incorporating CP yarns and UHMWPE braided yarns closely resembles traditional woven fabrics (Figure 5c), exhibiting excellent adaptability for textile applications. Under a stepwise current load (0.04 A → 0 A → 0.06 A → 0 A → 0.08 A → 0 A → 0.10 A), the fabric showed a continuous temperature increase over 1 min, surpassing the steady-state temperatures observed in single yarns. At 0.10 A, temperatures reached 72.3 °C for CP-8-20, 66.7 °C for CP-16-10, and 62.9 °C for CP-16-20.

In single-yarn tests, CP-16-10 demonstrated the highest steady-state temperature rise (Figure S8), which was expected due to the lack of thermal insulation from the UHMWPE sheath, allowing more direct heat transfer from the CNT core. However, in woven fabric, CP-8-20 exhibited the highest temperature rise, surpassing its performance as a single yarn (Table S2; Figure 5d,g). This can be attributed to the fabric’s role as a heat reservoir, enhancing the efficient transfer of heat from the internal CNTs through the UHMWPE to the external environment. CP-16-20, with its tightly braided structure and superior insulation, resulted in the lowest observed temperatures but ensured the most uniform temperature distribution across the fabric (Figure 5e). The loosely woven UHMWPE sheath of CP-16-10, which traps more static air, stores heat more effectively as a single yarn but shows only moderate temperatures when integrated into fabric due to reduced heat transfer (Figure 5f).

## 4. Conclusions

This work developed CP yarns with a breathable insulation layer and exceptional flexibility using a coaxial tubular braiding technique. All three CP yarns exhibited outstanding performance in toughness, abrasion, and wash resistance, as well as electrothermal properties. CP-16-20 offered the most complete core coverage, minimizing the risk of exposure, making it ideal for applications requiring high safety and protection. The CP-16-10 yarn, known for its superior flexibility, enhanced resistance stability under repetitive stress and is well-suited for applications requiring efficient Joule heating with fewer yarns. CP-8-20 demonstrated the highest electrothermal conversion efficiency in fabric, achieving a significant temperature increase from 25 °C to 72 °C at 0.1 A, making it ideal for textile-based heating applications. These optimized configurations advance the integration of insulation, flexibility, and thermal management, contributing to the development of next-generation wearable heating technologies. Furthermore, they hold potential for improving personal comfort and promoting energy efficiency, aligning with sustainability goals by supporting energy conservation and reducing carbon emissions.

## Data Availability

The original contributions presented in this study are included in the article/Supplementary Materials. Further inquiries can be directed to the corresponding authors.

## Supplementary Materials

The following supporting information can be downloaded at: https://www.mdpi.com/article/10.3390/polym17040537/s1, Figure S1: DSC curves of UHMWPE multifilaments; Figure S2: Digital images of UHMWPE filaments arranged in tightly packed two-layer and four-layer configurations; Figure S3: Infrared transmittance of UHMWPE filaments arranged in tightly packed configurations; Figure S4: EDOF image of the CNT yarn; Figure S5: Cross-sectional SEM images of the CP yarns; Figure S6: Stress-strain curves of the CNT yarns and CP yarns; Figure S7: EDOF images displaying the fracture morphology of the CNT and CP yarns; Figure S8:Temperature-time curves of the CNT and CP yarns during a single cycle of the cyclic electrothermal experiment, with current variations of 0 A → 0.05 A → 0.10 A → 0.05 A → 0 A; Figure S9: EDOF images of core-sheath CP yarns after folding; Figure S10: Digital images of the CNT yarns and CP yarns sewed on multi-fiber standard fabrics before and after washing; Table S1: Electrothermal performance of the CNT and CP yarns during a single cycle of the cyclic electrothermal experiment, with current variations of 0 A → 0.05 A → 0.10 A → 0.05 A → 0 A; Table S2: Electrothermal performance of three types of CP yarns and UHMWPE braided yarn fabrics during a cyclic electrothermal experiment with a current sequence of 0.04 A → 0 A → 0.06 A → 0 A → 0.08 A → 0 A → 0.10 A; Video S1: Embroidered “D” Component for Joule Heating via Near-Field Energy Reception.

## References

[1] Lee J., Llerena Zambrano B., Woo J., Yoon K., Lee T. (2020). Recent advances in 1D stretchable electrodes and devices for textile and wearable electronics: Materials, fabrications, and applications. Adv. Mater..

[2] Tseghai G.B., Malengier B., Fante K.A., Nigusse A.B., Van Langenhove L. (2020). Integration of conductive materials with textile structures, an overview. Sensors.

[3] Duan L.Y., D’hooge D.R., Cardon L. (2020). Recent progress on flexible and stretchable piezoresistive strain sensors: From design to application. Prog. Mater. Sci..

[4] Jia J., Pu J.H., Liu J.H., Zhao X., Ke K., Bao R.Y., Liu Z.Y., Yang M.B., Yang W. (2020). Surface structure engineering for a bionic fiber-based sensor toward linear, tunable, and multifunctional sensing. Mater. Horiz..

[5] Son W., Lee J.M., Kim S.H., Kim H.W., Cho S.B., Suh D., Chun S., Choi C. (2022). High-power hydro-actuators fabricated from biomimetic carbon nanotube coiled yarns with fast electrothermal recovery. Nano Lett..

[6] Wang C., Xia K., Wang H., Liang X., Yin Z., Zhang Y. (2019). Advanced carbon for flexible and wearable electronics. Adv. Mater..

[7] Li Q., Li K.R., Fan H.W., Hou C.Y., Li Y.G., Zhang Q.H., Wang H.Z. (2017). Reduced graphene oxide functionalized stretchable and multicolor electrothermal chromatic fibers. J. Mater. Chem. C.

[8] Luo J.C., Gao S.J., Luo H., Wang L., Huang X.W., Guo Z., Lai X.J., Lin L.W., Li R.K.Y., Gao J.F. (2021). Superhydrophobic and breathable smart MXene-based textile for multifunctional wearable sensing electronics. Chem. Eng. J..

[9] Pu J.H., Zhao X., Zha X.J., Bai L., Ke K., Bao R.Y., Liu Z.Y., Yang M.B., Yang W. (2019). Multilayer structured AgNW/WPU-MXene fiber strain sensors with ultrahigh sensitivity and a wide operating range for wearable monitoring and healthcare. J. Mater. Chem. A.

[10] Eom J., Jaisutti R., Lee H., Lee W., Heo J.S., Lee J.Y., Park S.K., Kim Y.H. (2017). Highly sensitive textile strain sensors and wireless user-interface devices using all-polymeric conducting fibers. ACS Appl. Mater. Interfaces.

[11] Pu J.H., Zha X.J., Zhao M., Li S., Bao R.Y., Liu Z.Y., Xie B.H., Yang M.B., Guo Z., Yang W. (2018). 2D end-to-end carbon nanotube conductive networks in polymer nanocomposites: A conceptual design to dramatically enhance the sensitivities of strain sensors. Nanoscale.

[12] Falco A., Loghin F.C., Becherer M., Lugli P., Salmeron J.F., Rivadeneyra A. (2019). Low-cost gas sensing: Dynamic self-compensation of humidity in CNT-based devices. ACS Sens..

[13] Zhu P., Liu Y., Fang Z., Kuang Y., Zhang Y., Peng C., Chen G. (2019). Flexible and highly sensitive humidity sensor based on cellulose nanofibers and carbon nanotube composite film. Langmuir.

[14] Tajin A.S., Levitt A.S., Liu Y., Amanatides C.E., Schauer C.L., Dion G., Dandekar K.R. (2020). On the effect of sweat on sheet resistance of knitted conductive yarns in wearable antenna design. IEEE AWPL.

[15] Walker J.M., Akbar S.A., Morris P.A. (2019). Synergistic effects in gas sensing semiconducting oxide nano-heterostructures: A review. Sens. Actuators B.

[16] Hossain M.M., Lubna M.M., Bradford P.D. (2023). Multifunctional and washable carbon nanotube-wrapped textile yarns for wearable e-textiles. ACS Appl. Mater. Interfaces.

[17] Li W., Xu F.J., Liu W., Gao Y., Zhang K., Zhang X.H., Qiu Y.P. (2018). Flexible strain sensor based on aerogel-spun carbon nanotube yarn with a core-sheath structure. Compos. Part A.

[18] Yang S., Liu S., Ding X.J., Zhu B., Shi J.D., Yang B., Liu S.R., Chen W., Tao X.M. (2021). Permeable and washable electronics based on polyamide fibrous membrane for wearable applications. Compos. Sci. Technol..

[19] Pan J.J., Hao B.W., Xu P.J., Li D.Q., Luo L., Li J.Q., Xia Z.G., Cheng D.S., Xu A.C., Cai G.M. (2020). Highly robust and durable core-sheath nanocomposite yarns for electro-thermochromic performance application. Chem. Eng. J..

[20] Yin Z., Jian M., Wang C., Xia K., Liu Z., Wang Q., Zhang M., Wang H., Liang X., Liang X. (2018). Splash-resistant and light-weight silk-sheathed wires for textile electronics. Nano Lett..

[21] Cai G., Yang M., Pan J., Cheng D., Xia Z., Wang X., Tang B. (2018). Large-scale production of highly stretchable CNT/cotton/spandex composite yarn for wearable applications. ACS Appl. Mater. Interfaces.

[22] Dong K., Peng X., An J., Wang A.C., Luo J., Sun B., Wang J., Wang Z.L. (2020). Shape adaptable and highly resilient 3D braided triboelectric nanogenerators as e-textiles for power and sensing. Nat. Commun..

[23] Gao Y., Li Z., Xu B., Li M., Jiang C., Guan X., Yang Y. (2022). Scalable core–spun coating yarn-based triboelectric nanogenerators with hierarchical structure for wearable energy harvesting and sensing via continuous manufacturing. Nano Energy.

[24] Sofronova D., Angelova R.A., Sofronov Y. (2021). Design and development of an e-textile mat for assuring the comfort of bedridden persons. Materials.

[25] Li Y.F., Liu H., Li X.J. (2017). Thermal-electrical properties and resistance stability of silver coated yarns. Adv. Mater. Mach. Electron. I.

[26] Dai H.M., Gao J.L., Jia C., Liu F.Y., Zhai G.X., Wang X.F., Xiang H.X., Zhu M.F. (2024). High-performance electrothermal fabrics enabled by lignin-derived carbon nanotube yarns. Chem. Eng. J..

[27] Yan J., Jeong Y.G. (2015). Highly elastic and transparent multiwalled carbon nanotube/polydimethylsiloxane bilayer films as electric heating materials. Mater. Des..

[28] Singh N., Sheikh J. (2022). Multifunctional linen fabric obtained through finishing with chitosan-gelatin microcapsules loaded with cinnamon oil. J. Nat. Fibers.

[29] Zuo X., Zhang X., Qu L., Miao J. (2023). Smart fibers and textiles for personal thermal management in emerging wearable applications. Adv. Mater. Technol..

[30] Kyosev Y. (2014). Braiding Technology for Textiles: Principles, Design and Processes.

[31] Gholami A., Melenka G.W. (2023). Studying the geometrical models of tubular braided composite using micro computed tomography and particle swarm optimization. Compos. Part. B-Eng..

[32] Zhai J., Zeng T., Xu G.D., Wang Z.H., Cheng S., Fang D.N. (2017). A multi-scale finite element method for failure analysis of three-dimensional braided composite structures. Compos. Part B.

[33] Liu Y.T., Hou Y.L., Sapanathan T., Nie R.J., Meng L., Xu Y.J. (2024). A multiscale strategy for exploring the mechanical behavior of 3D braided composite thin-walled cylinders. Thin Wall Struct..

[34] Wei X., Wang J., Ma H., Farha F.I., Bi S., Zhang Q., Xu F. (2022). Super-strong CNT composite yarn with tight CNT packing via a compress-stretch process. Nanoscale.

[35] Wu Y.D., Ma Q.Q., Liang T., Yao Y.M., Li J.H., Zeng X.L., Xu J.B., Sun R. (2022). A facile strategy to densify aligned CNT films with significantly enhanced thermal conductivity and mechanical strength. Adv. Mater. Technol..

[36] Zhan H., Chen Y.W., Shi Q.Q., Zhang Y., Mo R.W., Wang J.N. (2022). Highly aligned and densified carbon nanotube films with superior thermal conductivity and mechanical strength. Carbon.

[37] Tran T.Q., Fan Z., Liu P., Myint S.M., Duong H.M. (2016). Super-strong and highly conductive carbon nanotube ribbons from post-treatment methods. Carbon.

[38] Liu P., Hu D.C.M., Tran T.Q., Jewell D., Duong H.M. (2016). Electrical property enhancement of carbon nanotube fibers from post treatments. Colloid. Surface A.

[39] Dini Y., Rouchon D., Faure-Vincent J., Dijon J. (2020). Large improvement of CNT yarn electrical conductivity by varying chemical doping and annealing treatment. Carbon.

[40] Taylor L.W., Dewey O.S., Headrick R.J., Komatsu N., Peraca N.M., Wehmeyer G., Kono J., Pasquali M. (2021). Improved properties, increased production, and the path to broad adoption of carbon nanotube fibers. Carbon.

[41] Gao E.L., Lu W.B., Xu Z.P. (2018). Strength loss of carbon nanotube fibers explained in a three-level hierarchical model. Carbon.

[42] Jung Y., Cho Y.S., Lee J.W., Oh J.Y., Park C.R. (2018). How can we make carbon nanotube yarn stronger?. Compos. Sci. Technol..

[43] Wang J.N., Luo X.G., Wu T., Chen Y. (2014). High-strength carbon nanotube fibre-like ribbon with high ductility and high electrical conductivity. Nat. Commun..

