# Supplementary material for: Enhanced Electrothermal Properties of Core–Sheath Lignin-Derived Carbon Nanotube Yarns with UHMWPE Insulation
Source: Polymers (Basel). 2025 Feb 19;17(4):537. doi: 10.3390/polym17040537 (PMC11860152; doi:10.3390/polym17040537)
Supplement: Supplementary file 1 [file polymers-17-00537-s001.zip › SI 2025 0218.pdf]

Supporting Information

Enhanced Electrothermal Properties of Core–Sheath Lignin-Derived Carbon Nanotube Yarns with UHMWPE Insulation

Hongmei Dai, Chao Jia *, Zexu Hu, Senlong Yu, Hengxue Xiang *, Xuefen Wang and Meifang Zhu

State Key Laboratory of Advanced Fiber Materials, College of Materials Science and Engineering, Donghua University, Shanghai 201620, China; 1209738@mail.dhu.edu.cn (H.D.); huzexu@dhu.edu.cn (Z.H.); ysl@dhu.edu.cn (S.Y.); wangxf@dhu.edu.cn (X.W.); zmf@dhu.edu.cn (M.Z.)

* Correspondence: jiachao0806@dhu.edu.cn (C.J.); hengxuexiang@dhu.edu.cn (H.X.);

Tel.: +86-21-67792849 (C.J. & H.X.); Fax: +86-2-67792855 (C.J. & H.X.)

Supplementary Figures:

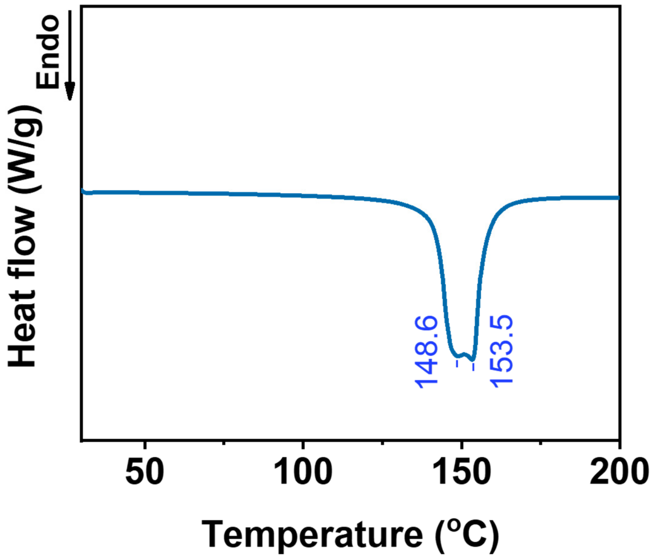

Figure S1. DSC curves of UHMWPE multifilaments.

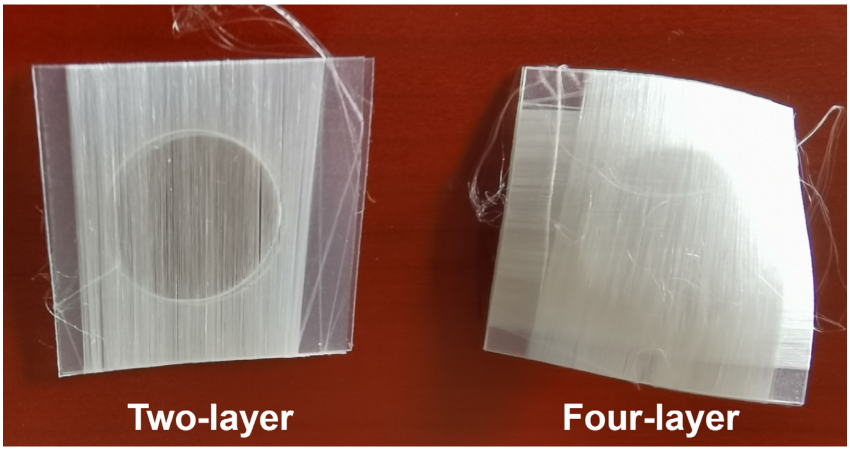

Figure S2. Digital images of UHMWPE filaments arranged in tightly packed two-layer and four-layer configurations.

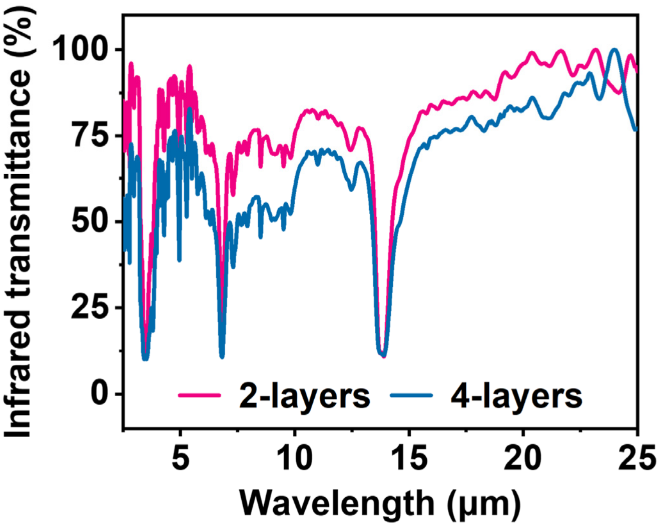

Figure S3. Infrared transmittance of UHMWPE filaments arranged in tightly packed configurations.

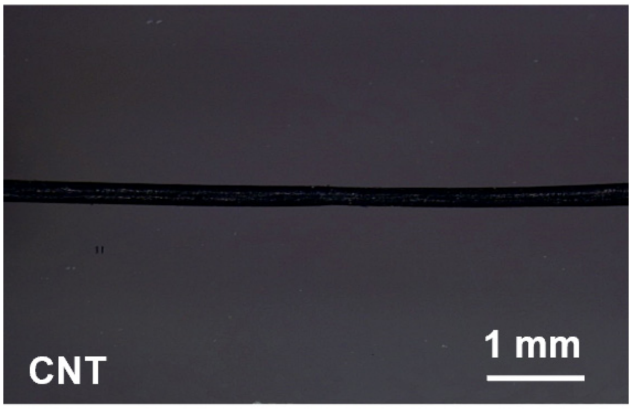

Figure S4. EDOF image of the CNT yarn.

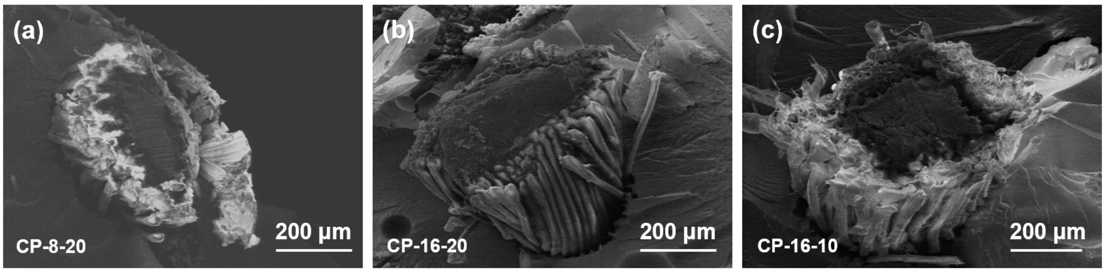

Figure S5. Cross-sectional SEM images of the CP yarns. (a) CP-8-20, (b) CP-16-20, and (c) CP-16-10.

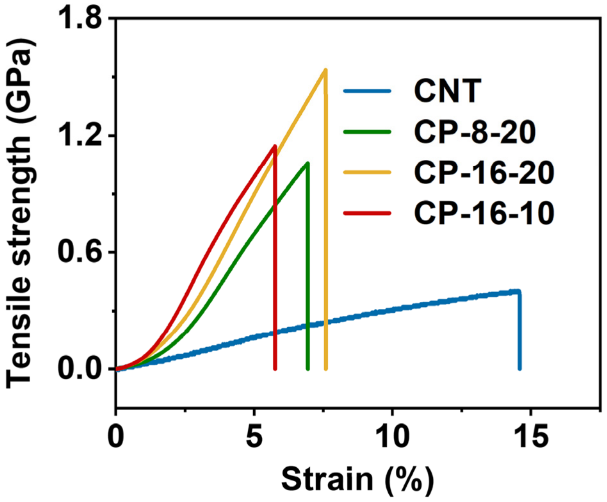

Figure S6. Stress-strain curves of the CNT yarns and CP yarns.

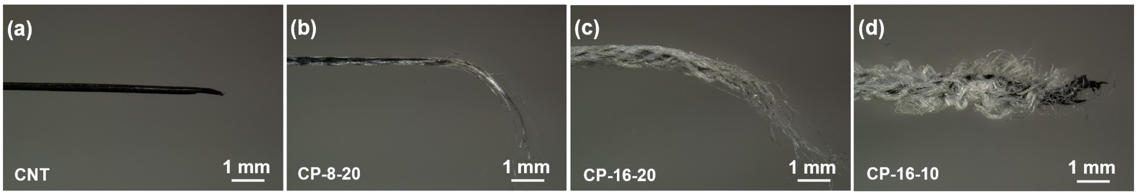

Figure S7. EDOF images displaying the fracture morphology of the CNT and CP yarns. (a) CNT yarn, (b) CP-8-20, (c) CP-16-20, and (d) CP-16-10.

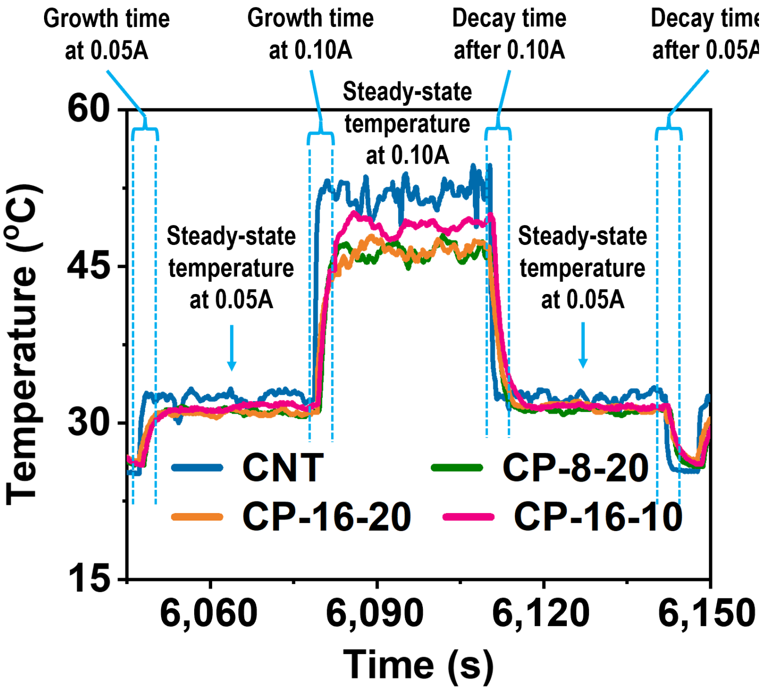

Figure S8. Temperature-time curves of the CNT and CP yarns during a single cycle of the cyclic electrothermal experiment, with current variations of 0 A→0.05 A→0.10 A→0.05 A→0 A.

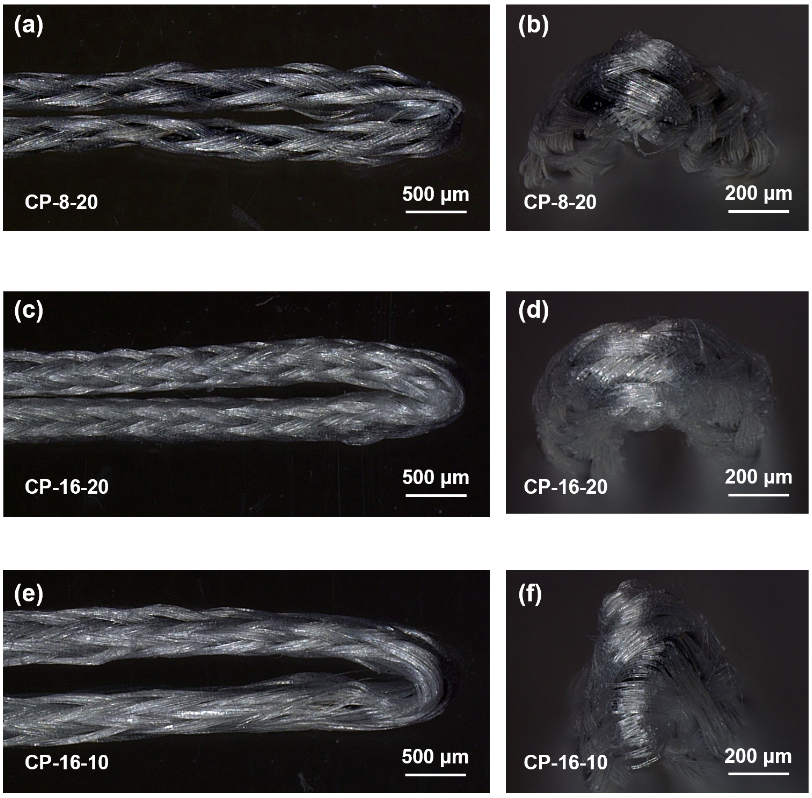

Figure S9. EDOF images of core-sheath CP yarns after folding. (a-b) CP-8-20. (c-d) CP-16-20. (e-f) CP-16-10.

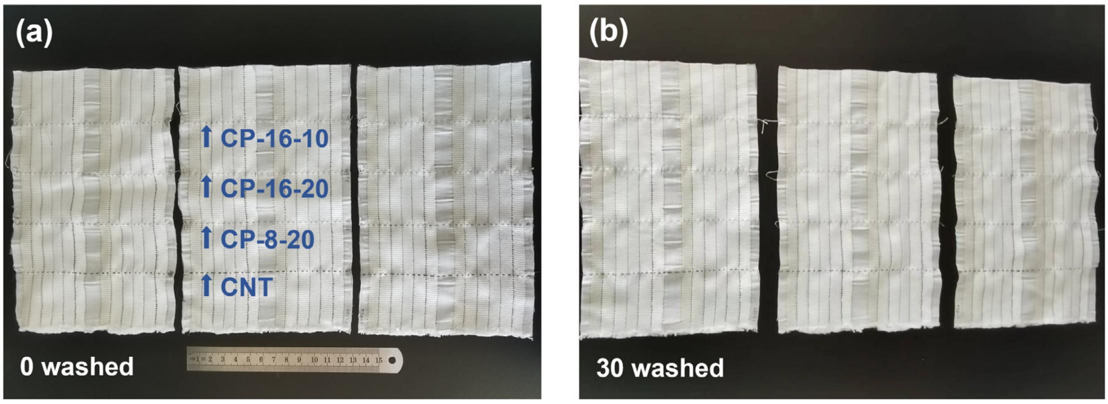

Figure S10. Digital images of the CNT yarns and CP yarns sewed on multi-fiber standard fabrics (a) before and (b) after washing.

Supplementary Tables:

Table S1. Electrothermal performance of the CNT and CP yarns during a single cycle of the cyclic electrothermal experiment, with current variations of 0 A→0.05 A→0.10 A→0.05 A→0 A.

**Sample**	**Current**	τ_g	τ_d	h_{r+c}
	**A**	**s**	**s**	**mW °C^{-1} cm^{-1}**
CNT yarn	0.05	0.38±0.00	0.32±0.00	1.18
	0.10	0.32±0.03	0.29±0.02	1.24
CP-8-20	0.05	1.18±0.06	1.08±0.05	1.69
	0.10	1.28±0.09	1.01±0.06	1.71
CP-16-20	0.05	1.75±0.05	1.75±0.05	1.73
	0.10	1.75±0.16	1.46±0.10	1.72
CP-16-10	0.05	1.64±0.12	1.59±0.13	1.59
	0.10	1.59±0.07	1.52±0.06	1.59

Table S2. Electrothermal performance of three types of CP yarns and UHMWPE braided yarn fabrics during a cyclic electrothermal experiment with a current sequence of 0.04 A→0 A→0.06 A→0 A→0.08 A→0 A→0.10 A.

Sample	Current	CP-3mm @ 10×10 cm			
	A	τ_g s	τ_d s	h_{r+c} mW $^oC^{-1}$ cm^{-2}	h_{r+c} mW ^oC pcs^{-1} cm^{-1}
CP-8-20	0.04	5.34	5.64	4.4	1.1
	0.06	6.25	6.30	5.4	1.2
	0.08	5.55	6.75	5.5	1.3
	0.10	5.76	5.83	5.2	1.2
CP-16-20	0.04	7.87	6.57	4.5	1.3
	0.06	6.99	6.96	5.3	1.4
	0.08	5.82	5.41	6.3	1.6
	0.10	6.39	5.89	6.2	1.6
CP-16-10	0.04	6.13	4.59	5.5	1.4
	0.06	6.18	6.16	5.4	1.2
	0.08	6.94	6.40	6.5	1.5
	0.10	5.80	5.92	6.2	1.4
